# Synthesis of Morphinan Alkaloids in *Saccharomyces cerevisiae*

**DOI:** 10.1371/journal.pone.0124459

**Published:** 2015-04-23

**Authors:** Elena Fossati, Lauren Narcross, Andrew Ekins, Jean-Pierre Falgueyret, Vincent J. J. Martin

**Affiliations:** 1 Department of Biology, Concordia University, Montréal, Québec, Canada; 2 Centre for Structural and Functional Genomics, Concordia University, Montréal, Québec, Canada; CNR, ITALY

## Abstract

Morphinan alkaloids are the most powerful narcotic analgesics currently used to treat moderate to severe and chronic pain. The feasibility of morphinan synthesis in recombinant *Saccharomyces cerevisiae* starting from the precursor (*R*,*S*)-norlaudanosoline was investigated. Chiral analysis of the reticuline produced by the expression of opium poppy methyltransferases showed strict enantioselectivity for (*S*)-reticuline starting from (*R*,*S*)-norlaudanosoline. In addition, the *P*. *somniferum* enzymes salutaridine synthase (PsSAS), salutaridine reductase (PsSAR) and salutaridinol acetyltransferase (PsSAT) were functionally co-expressed in *S*. *cerevisiae* and optimization of the pH conditions allowed for productive spontaneous rearrangement of salutaridinol-7-*O*-acetate and synthesis of thebaine from (*R*)-reticuline. Finally, we reconstituted a 7-gene pathway for the production of codeine and morphine from (*R*)-reticuline. Yeast cell feeding assays using (*R*)-reticuline, salutaridine or codeine as substrates showed that all enzymes were functionally co-expressed in yeast and that activity of salutaridine reductase and codeine-*O*-demethylase likely limit flux to morphine synthesis. The results of this study describe a significant advance for the synthesis of morphinans in *S*. *cerevisiae* and pave the way for their complete synthesis in recombinant microbes.

## Introduction

**Fig 1:**
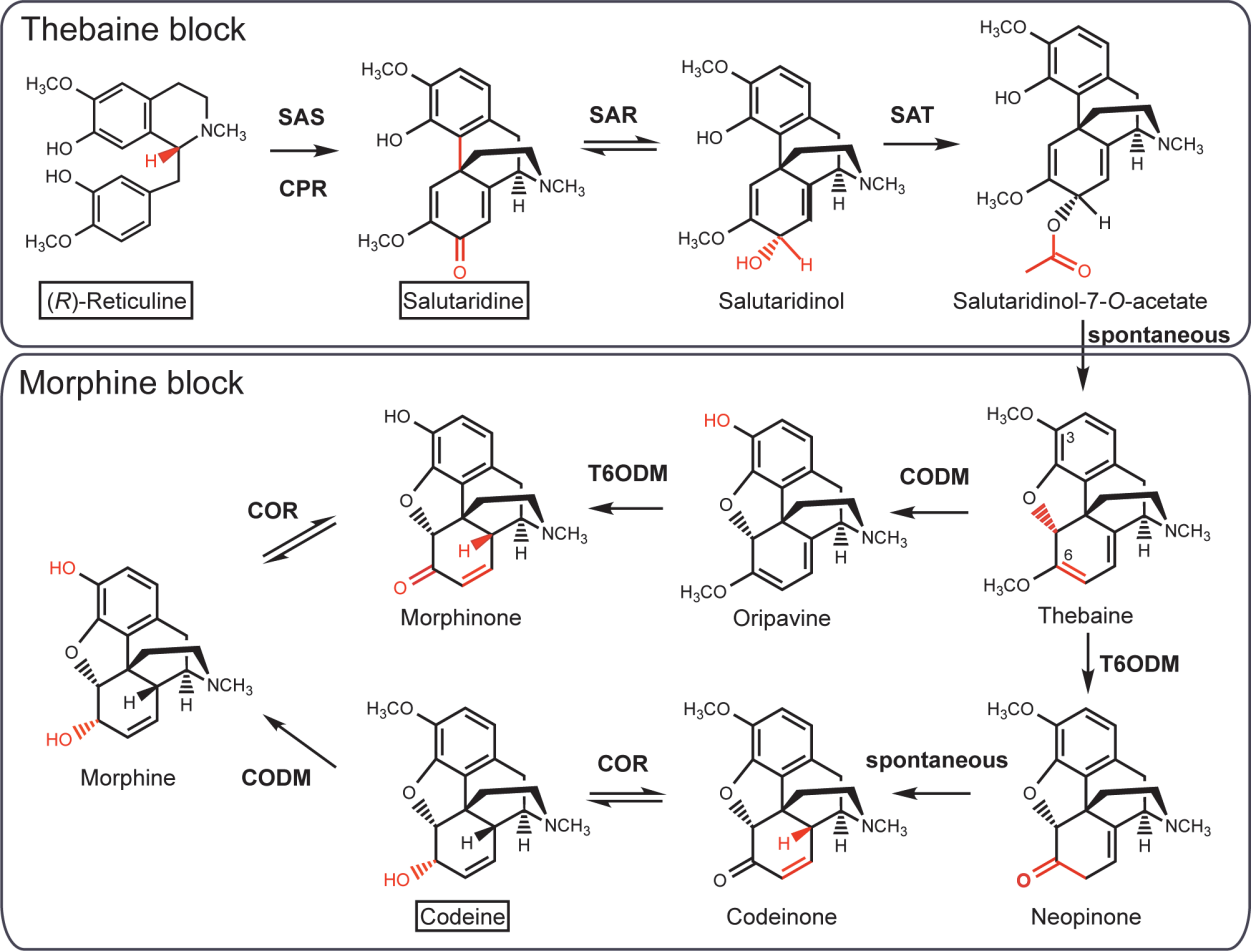
Description of the (*R*)-reticuline to morphine biosynthetic pathway reconstituted in *S*. *cerevisiae*. The pathway is divided in two blocks of sequential enzymes all from *P*. *somniferum*. The thebaine block includes the enzymes involved in the synthesis of thebaine from (*R*)-reticuline: PsSAS, salutaridine synthase; PsCPR, cytochrome P450 reductase; PsSAR, salutaridinol reductase; PsSAT, salutaridinol acetyltransferase. The morphine block is composed of enzymes involved in the synthesis of morphine from thebaine: PsT6ODM, thebaine 6-*O*-demethylase; PsCOR, codeinone reductase; PsCODM, codeine-*O*-demethylase. Boxed text identifies intermediates used as feeding substrates to test for functional expression of the assembled pathways in yeast.

Morphinan alkaloids are the most powerful narcotic analgesics currently used to treat moderate to severe and chronic pain. They include the opiates codeine and morphine and their semi-synthetic derivatives, such as dihydromorphine and hydromorphone. The opioids antagonist naloxone and naltrexone, used to treat opiate addiction and overdose are derived from thebaine. Thebaine is a precursor to codeine and morphine biosynthesis *in planta* (Fig 1) and is also the starting precursor for the chemical synthesis of the analgesics oxycodone and buprenorphine, which have more favourable side-effect profiles than morphine [1,2].

Cultivars of opium poppy improved for optimal opiate production by extensive breeding cycles and mutagenesis are the only commercial source of thebaine and morphine [2,3]. While the supply of morphinan alkaloids from plant extraction currently meets demand [3], microbial production systems for BIAs and other alkaloids are being developed in order to provide alternative sources [4–6]. Efficient production of opiates using microbial platforms could not only contribute to reduce the cost of opiate production, but also offer a versatile platform for the creation of new scaffolds for drug discovery [7]. This refers to both alkaloids that do not accumulate in sufficient quantity and new molecules not yet isolated nor produced from plants.

**Fig 2:**
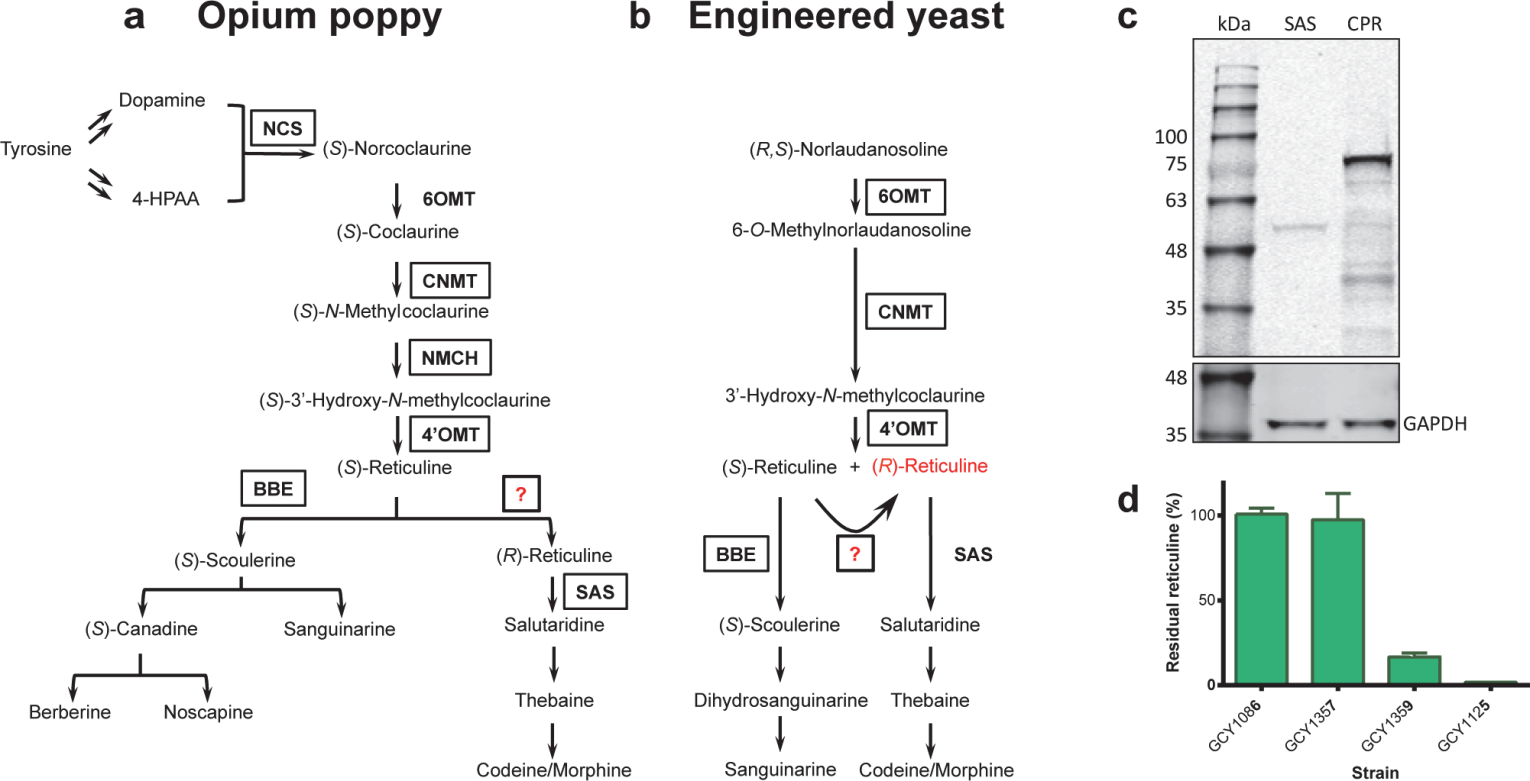
Reticuline production and utilization in engineered *S*. *cerevisiae*. Biochemical pathway depicting reticuline production and utilization in **(a)** opium poppy and **(b)** engineered *S*. *cerevisiae*. **(c)** Immunoblot analysis of recombinant PsSAS (56 kDa) and PsCPR (76 kDa) expression in *S*. *cerevisiae*. GAPDH (36 kDa) was used as a loading control. **(d)** Reticuline production and utilization by strains expressing a recombinant reticuline-producing pathway (strain GCY1086) as well as PsSAS (strain GCY1357), PsBBE (strain GCY1359) or a complete dihydrosanguinarine pathway (strain GCY1125; [4]).

Morphinan alkaloids belong to a broader class of plant secondary metabolites known as benzylisoquinoline alkaloids (BIAs), with diverse pharmaceutical properties including the muscle relaxant papaverine, the antimicrobials berberine and sanguinarine and the antitussive and potential anticancer drug noscapine [8,9]. Thousands of distinct BIAs have been identified in plants, all derived from a single precursor: (*S*)-norcoclaurine. BIA synthesis in plants proceeds through the enantioselective Pictet-Spengler condensation of the L-tyrosine derivatives L-dopamine and 4-hydroxyphenylacetaldehyde to produce (*S*)-norcoclaurine, catalyzed by the enzyme norcoclaurine synthase (NCS; Fig 2A) [10]. (*S*)-Norcoclaurine can be converted to the branch point intermediate (*S*)-reticuline via one hydroxylation and three methylation events (Fig 2A). In *P*. *somniferum* the morphine pathway diverges from other BIA pathways in that it proceeds through (*R*)-reticuline instead of (*S*)-reticuline (Fig 2A). The epimerization of (*S*)-reticuline to (*R*)-reticuline has been proposed to proceed via dehydrogenation of (*S*)-reticuline to 1,2-dehydroreticuline by dehydroreticuline synthase (DRS) and subsequent enantioselective reduction to (*R*)-reticuline by dehydroreticuline reductase (DRR). While the corresponding enzymes have been purified from *P*. *somniferum* seedlings, the genes encoding these enzymes have never been cloned and their products have not been fully characterized [11,12]. However, it is believed that the three *P*. *somniferum* methyltransferases (MTs) Ps6OMT, PsCNMT and Ps4’OMT, can synthesize racemic reticuline from commercial (*R*,*S*)-norlaudanosoline in *S*. *cerevisiae* [13], thereby bypassing the need for the (*S*)- to (*R*)-reticuline epimerization step of morphinan biosynthesis. (*R*,*S*)-Norlaudanosoline synthesis from glycerol in *E*. *coli* has recently been achieved at 287 mg/L, the highest yields for a microbially-synthesized BIA reported to date, increasing (*R*,*S*)-norlaudanosoline’s attractiveness as a starting point for microbial BIA synthesis [14].

The synthesis of the BIA branch point intermediate reticuline has been achieved from simple carbon sources in *E*. *coli* [14] and from (*R*,*S*)-norlaudanosoline in *S*. *cerevisiae* [13]. Previously, we reported on the biosynthesis of dihydrosanguinarine from (*R*,*S*)-norlaudanosoline by co-expressing 10 genes in *S*. *cerevisiae* [4]. Recently, production of morphine and semi-synthetic opioids from thebaine was also reported in *S*. *cerevisiae* [5]. Despite these significant advances, major challenges will have to be addressed before a viable microbial process for morphinan synthesis can be reached, including reconstituting a complete BIA pathway starting from a low cost substrate and optimization of yields.

In the current study, we investigated the feasibility of morphinan synthesis in yeast from (*R*,*S*)-norlaudanosoline. Chiral analysis of the reticuline produced by the opium poppy methyltransferases expressed in yeast demonstrated that (*R*)-reticuline cannot be generated from (*R*)-norlaudanosoline and hence the proposed epimerization step remains key to assembling a complete functional heterologous morphinan pathway in microbes. In addition, the *P*. *somniferum* enzymes salutaridine synthase (PsSAS), salutaridine reductase (PsSAR) and salutaridinol acetyltransferase (PsSAT) (Fig 1) were functionally co-expressed in *S*. *cerevisiae* and optimization of pH conditions allowed for productive spontaneous rearrangement of salutaridinol-7-*O*-acetate and synthesis of thebaine from (*R*)-reticuline. Finally, we reconstituted a 7-gene pathway for the production of codeine and morphine from supplemented precursors in *S*. *cerevisiae*. The results of this study describe a significant advance for the synthesis of morphinans in *S*. *cerevisiae* and pave the way for their complete synthesis in recombinant microbes.

## Materials and Methods

### Chemicals and reagents

(*S*)-Reticuline was a gift from Peter Facchini (University of Calgary). (*R*,*S*)-Norlaudanosoline was purchased from Enamine Ltd. (Kiev, Ukraine), (*R*)-reticuline, salutaridine, thebaine, oripavine, codeine and morphine were from TRC Inc. (North York, Ontario, Canada). Antibiotics, growth media and α-D-glucose were purchased from Sigma-Aldrich. Restriction endonuclease enzymes were from New England Biolabs (NEB). Yeast genomic DNA used as template for PCR was purified using the DNeasy Blood and Tissue Kit (Qiagen). Polymerase chain reactions (PCRs) were performed using Phusion High-Fidelity DNA polymerase (NEB/Thermo Scientific). PCR-amplified products were gel purified using the QIAquick Purification Kit (Qiagen). Plasmid extractions were done using the GeneJET plasmid mini-prep kit (Thermo Scientific). HPLC-grade water was purchased from Fluka. HPLC-grade acetonitrile was purchased from Fischer Scientific.

### Plasmids and *S*. *cerevisiae* strains construction

All enzymes used in this work are from *P*. *somniferum*. Synthetic sequences of *6OMT* (GenBank KF554144), *4’OMT* (corresponding to 4’OMT2, GenBank KF661327), *CNMT* (GenBank KF661326), *P450R (CPR)* (GenBank KF661328), *SAS* (GenBank KP400664), *SAR* (GenBank KP400665), *SAT* (GenBank KP400666), *CODM* (GenBank KP400667), *T6ODM* (GenBank KP400668) and *COR* (corresponding to COR1.3, GenBank KP400669) were codon-optimized by DNA2.0 (Menlo Park, CA) for optimal expression in yeast. The partial Kozak sequence AAAACA was introduced upstream of all coding sequences as an integral part of gene synthesis.

Plasmids sequences were designed to independently express sequential enzymes of the morphine pathway (Table 1). The enzymes were cloned into the pGREG series of *E*. *coli*-*S*. *cerevisiae* shuttle vectors [15]. Modified vectors pGREG503, 504, 505 and 506, harbouring respectively the *HIS3*, *TRP1*, *LEU2* and *URA3* auxotrophic markers and containing a unique *Kpn*I site were used [4]. Gene expression cassettes were inserted by homologous recombination into pGREG vectors previously linearized with *Asc*I/*Kpn*I. Empty pGREG control plasmids created by intra-molecular ligation of the linearized pGREG made blunt with T4 DNA polymerase were used as negative controls [4]. *In vivo* homologous recombination in yeast was used for assembly of the plasmids [16]. Promoters, genes and terminators were assembled by incorporating a ~50-bp homologous region between the segments. DNA linkers (C6-H(n)-C1) were used to join cassettes to each other and to the vector backbone as well as to join each gene to its terminator in plasmid pGC359 (S1 Table and S2 Table). Promoters and terminators were amplified using CEN.PK genomic DNA as template. Primers used to amplify assembly parts are described in S3 Table.

**Table 1:** List of *S*. *cerevisiae* strains and plasmids used in this study.

Strain	Plasmids^a^ ^,^ ^b^	Source
GCY256	pGC263 (SAS-HA tag)	This study
GCY257	pGC264 (CPR-HA tag)	This study
GCY258	pGC265 (SAR-HA tag)	This study
GCY368	pGC359 (SAS, CPR, SAR, SAT)	This study
GCY1086	pGC1062 (6OMT, 4’OMT, CNMT)	[4]
GCY1125 (dihydrosanguinarine producing strain)	pGC1062 (6OMT, CNMT, 4’OMT); pGC557 (CPR); pGC655 (BBE)	[4]
GCY1356	pGC719 (SAS, CPR)	This study
GCY1357	pGC1062 (6OMT, CNMT, 4’OMT); pGC719 (SAS, CPR)	This study
GCY1358	pGC359 (SAS, CPR, SAR, SAT); pGC11 (CODM, T6ODM, COR)	This study
GCY1359	pGC1062 (6OMT, CNMT, 4’OMT); pGC655 (BBE)	This study

Full genotypes are available in Supporting Information S1 Table.

^a^ All the genes used in this study are synthetic genes and sequences were codon-optimized for expression in *Saccharomyces cerevisiae*.

^b^ All protein sequences are from *Papaver somniferum*.

PsSAS, PsCPR and PsSAR were also independently cloned as HA-tagged constructs into the 2μ vector pYES2 (Table 1). For DNA assembly, the pYES2 backbone was amplified by PCR using primers pYES2 for and pYES2 rev. All primers used to modify pYES2 are described in S3 Table. Transformation of DNA fragments in yeast for homologous recombination was accomplished by electroporation in the presence of sorbitol [16].

All plasmids assembled in yeast were transferred to *E*. *coli* and sequenced-verified. Yeast strains for opiate production were obtained by transformations of plasmids using heat shock in the presence of lithium acetate, carrier DNA and PEG 3350 [17]. Yeast nitrogen broth supplemented with synthetic dropout, 2% α-D-glucose (SC-GLU) and 2% agar was used for selection of plasmid transformation on solid media. For liquid cultures, *S*. *cerevisiae* was grown SC-GLU at 30°C and 200 rpm. All plasmids and yeast strains used in this work are described in Table 1 and S1 Table.

### Immunoblot analysis of PsSAS and PsCPR

Yeast strains GCY256 and GCY257 (Table 1) expressing HA-tagged PsSAS and PsCPR respectively, were grown overnight in SC medium with 0.2% glucose and 1.8% galactose as carbon sources. Ten ml of fresh medium containing 2% galactose as a carbon source was inoculated with 5% of the overnight cultures and incubated at 30°C and 200 rpm. Cells were harvested by centrifugation at OD_600_ of approximately 0.6 and lysed by bead beating in IP150 buffer (50 mM Tris-HCl (pH 7.4), 150 mM NaCl, 2 mM MgCl2, 0.1% Nonidet P-40) supplemented with cOmplete Mini Protease Inhibitor Cocktail (Roche Applied Science). The lysates were cleared by centrifugation and protein concentration was estimated using a Coomassie protein assay reagent (Thermo Scientific). Forty μg of total protein extract were resolved by SDS-PAGE and transferred to nitrocellulose for detection of the HA epitope using mouse anti-HA tag antibody HA-C5 (Abcam). As a loading control, glyceraldehyde-3-phosphate dehydrogenase (GAPDH) was detected using rabbit anti-GAPDH (Rockland Immunochemicals). Imaging of the blot was performed using an Odyssey imager and IRDye secondary antibodies (LI-COR Biosciences).

### Cell feeding assays

Whole cell substrate feeding assays were used to test the function of enzymes individually and in combination. Assays were performed in 96-well plates. A colony of *S*. *cerevisiae* was inoculated in 100 μl of SC-GLU and incubated for 24 hrs on a rotatory shaker at 30°C and 400 rpm. Cultures were diluted to 10% into 1 ml of fresh SC-GLU and incubated for an additional 6 hrs. Cells were harvested by centrifugation at 2000 x *g* for 2 min. Supernatants were decanted and cells were suspended in 300 μl of 100 mM Tris-HCl pH 7.5, 8, 8.5 or 9, containing 100 μM of the appropriate substrate: (*R*,*S*)-norlaudanosoline, (*R*)-reticuline, salutaridine or codeine. Cells were incubated for 16 to 20 hrs and then harvested by centrifugation at 4000 x *g* for 1 min. For BIA extraction from cells, the cell pellet was suspended in 300 μl methanol and vortexed for 30 min. Cell extracts were clarified by centrifugation at 4000 x *g* for 1 min and used directly for LC-MS analysis. Supernatant fractions were diluted in methanol to keep alkaloid concentrations within the range of standard curve values and to avoid saturating FT signals. As negative controls, yeast strains transformed with the correspondent empty plasmids were incubated with each of the pathway intermediates.

### Chiral analysis of reticuline

Separation of the (*R*)- and (*S*)- enantiomers of reticuline was performed using the chiral column CHIRALCEL OD-H (4.6x250mm, Daicel Chemical Industries) and the solvent system hexane:2-propanol:diethylamine (78:22:0.01) at a flow rate of 0.55 ml min^-1^ [18]. Following LC separation, metabolites were injected into an LTQ ion trap mass spectrometer (Thermo Electron, San Jose, CA) and detected by selected reaction monitoring (SRM). SRM transitions of m/z 288**→**164.0 (CID@35) and 330**→**192 (CID@30) were used to detect reticuline. Retention times for reticuline obtained in samples matched retention times observed with authentic standards.

### FT-MS analysis of alkaloids

Detection of opiates in the morphine biosynthetic pathway was performed by FT-MS using a 7T-LTQ FT ICR instrument (Thermo Scientific, Bremen, Germany). Alkaloids were separated by reverse phase HPLC (Perkin Elmer SERIES 200 Micropump, Norfolk, CT) using an Agilent Zorbax Rapid Resolution HT C18 2.1x30 mm, 1.8 micron column. Solvent A (0.1% formic acid) and solvent B (100% acetonitrile, 0.1% formic acid) were used in a gradient elution to separate the metabolites of interest as follows: 0–1 min at 100% A, 1–6 min 0 to 95% B (linear gradient), 7–8 min 95% B, 8–8.2 min 100% A, followed by a 1 min equilibration at 100% A. Five μl of either cell extract or supernatant fraction were loaded on the HPLC column run at a constant flow rate of 0.25 ml/min. Following LC separation, metabolites were injected into the LTQ-FT-MS (ESI source in positive ion mode) using the following parameters: resolution, 100000; scanning range, 250 to 450 AMU; capillary voltage, 5 kV; source temperature, 350°C; AGC target setting for full MS were set at 5 x 10^5^ ions. Identification of alkaloids was done using retention time and exact mass (<2 ppm) of the monoisotopic mass of the protonated molecular ion [M + H]^+^. Authentic standard of (*R*)-reticuline, (*S*)-reticuline, salutaridine, thebaine, oripavine, codeine and morphine were used to confirm the identity of BIA intermediates and to quantify morphine alkaloids. MS2 spectra were also used to further validate compound identity. When unavailable, we assumed equal ionization efficiency between an intermediate and the closest available quantifiable alkaloid: salutaridinol (*m/z* = 330) as salutaridine, codeinone (*m/z* = 298) and neopine (*m/z* = 300) as codeine.

## Results

### Reticuline production and utilization in engineered *S*. *cerevisiae*

Opium poppy salutaridine synthase (CYP719B1; PsSAS), the enzyme converting (*R*)-reticuline to salutaridine, has been characterized as strictly enantioselective for the (*R*)-enantiomer of reticuline [19]. However, salutaridine synthesis from (*R*,*S*)-norlaudanosoline has been achieved in yeast using the 3 opium poppy MTs and the human cytochrome P450 enzyme CYP2D6 as a surrogate source of salutaridine synthase [13]. Unfortunately the enantioselectivity of CYP2D6 was not reported. In the current study, the opium poppy enantioselective PsSAS was used for the reconstitution of the morphinan pathway in yeast (Fig 2B). While expression in yeast of the opium poppy salutaridine synthase and its cognate reductase could be confirmed by immunoblotting (Fig 2C), PsSAS activity as measured by salutaridine production or reticuline consumption in a reticuline-producing strain supplemented with (*R*,*S*)-norlaudanosoline was not detected (strains GCY1086 and GCY1357; Fig 2D). In contrast, when co-expressing the three *P*. *somniferum* MTs (Ps6OMT, PsCNMT and Ps4’OMT) with the berberine bridge enzyme (PsBBE), which is enantioselective for the (*S*)-enantiomer of reticuline [20], greater than 50% consumption of the reticuline produced by yeast cells was observed (strain GCY1359; Fig 2D). Furthermore, near complete consumption of the reticuline intermediate was observed using a dihydrosanguinarine-producing strain, allegedly due to a pull on reticuline from the downstream pathway (strain GCY1125; Fig 2D). Taken together, the lack of reticuline turnover by the PsSAS expressing strain and the near complete utilisation of reticuline in a dihydrosanguinarine-producing strain suggested that the reticuline being produced from (*R*,*S*)-norlaudanosoline was not racemic but was in fact only the (*S*)-enantiomer.

### Chiral analysis of reticuline produced in *S*. *cerevisiae*

**Fig 3:**
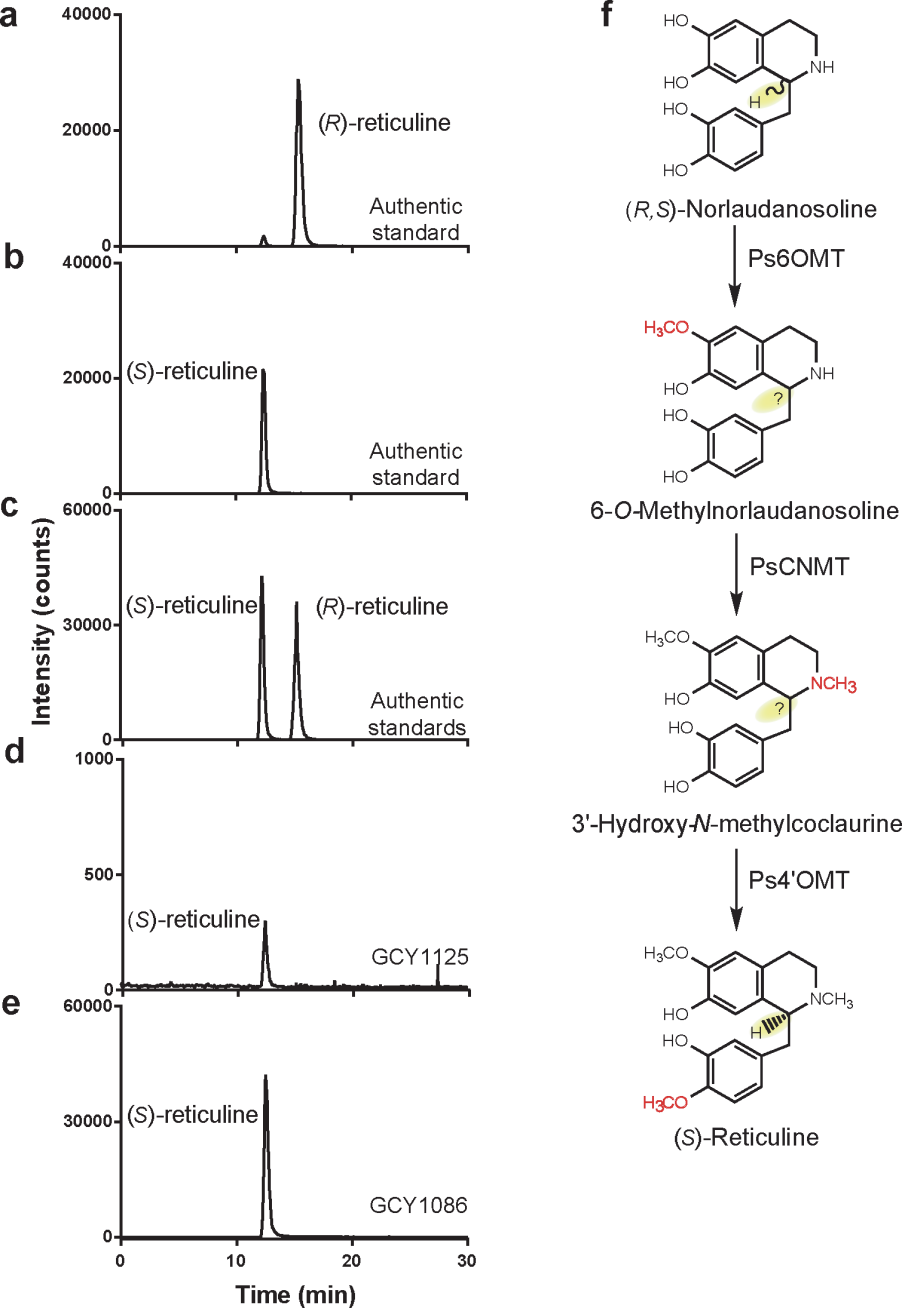
Chiral analysis of reticuline produced from (*R*,*S*)-norlaudanosoline by engineered *S*. *cerevisiae*. HPLC-MS chromatographic profile of authentic standards of **(a)** (*R*)-reticuline, **(b)** (*S*)-reticuline and **(c)** a mixture of (*S*)- and (*R*)-reticuline. **(d)** Chiral analysis of reticuline produced from (*R*,*S*)-norlaudanosoline in cell feeding assays of strain GCY1125 expressing the opium poppy Ps6OMT, PsCNMT, Ps4’OMT and a complete dihydrosanguinarine pathway [4]. **(e)** Chiral analysis of reticuline produced from (*R*,*S*)-norlaudanosoline in cell feeding assays of strain GCY1086 expressing the opium poppy Ps6OMT, PsCNMT and Ps4’OMT. **(f)** Methylation pathway for conversion of (*R*,*S*)-norlaudanosoline to (*S*)-reticuline.

To investigate the possibility that the reticuline produced from (*R*,*S*)-norlaudanosoline by the three opium poppy MTs was not racemic, chiral analysis by HPLC was used to reveal the presence or absence of reticuline enantiomers. Chiral analysis of enantio-pure standards of (*R*)- and (*S*)-reticuline and of racemic (*R*,*S*)-reticuline was first performed to confirm the separation of the two enantiomers (Fig 3A, 3B and 3C). Analysis of the reticuline produced by the BBE-expressing strain GCY1125, which was assumed to accumulate (*R*)-reticuline and convert (*S*)-reticuline into scoulerine, showed instead that only trace (*S*)-reticuline remained (Figs 2D and 3D). Finally, chiral analysis of the reticuline produced by strain GCY1086 expressing only the three *P*. *somniferum* MTs revealed that only (*S*)-reticuline was produced (Fig 3E), demonstrating the strict enantioselectivity of one or more of the three MTs on racemic norlaudanosoline (Fig 3F).

### Functional expression of PsSAS in *S*. *cerevisiae*

**Fig 4:**
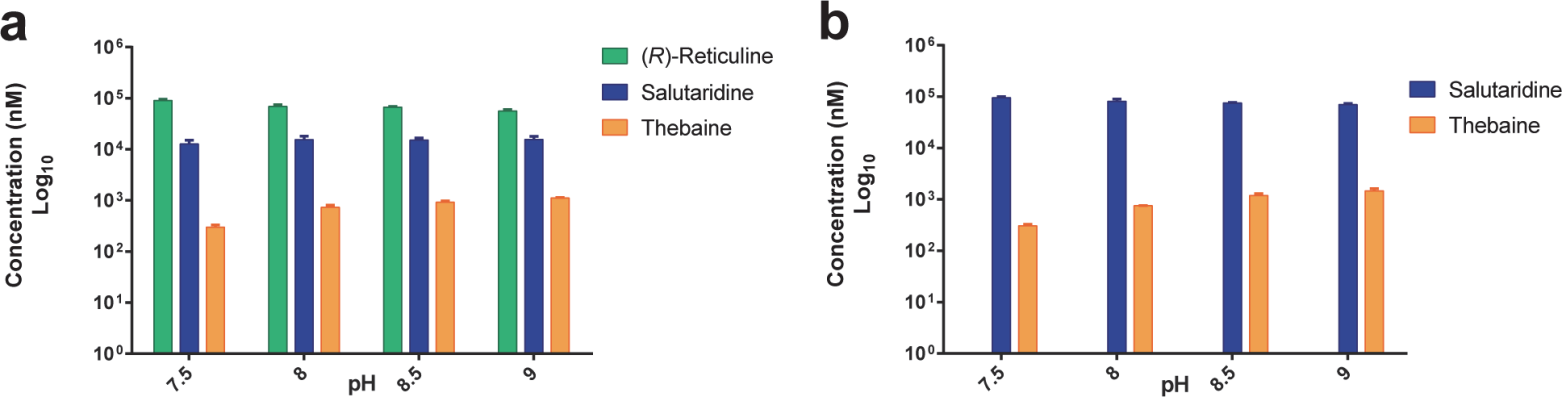
Synthesis of thebaine in engineered *S*. *cerevisiae*. Synthesis of thebaine from cell feeding assays at pH 7.5, 8, 8.5 or 9 and supplemented with **(a)** 100 μM (*R*)-reticuline or **(b)** 100 μM salutaridine. Strain GCY368 was used in all cell feeding assays. Error bars represent standard deviations of n = 3.

The enzyme salutaridine synthase has been characterized from its heterologous expression and purification from insect cells [19]. PsSAS can accept both (*R*)-reticuline and (*R*)-norreticuline as substrates, but not their corresponding (*S*)-enantiomers. The functional expression of PsSAS in *S*. *cerevisiae* was tested using cell feeding assays supplemented with (*R*)-reticuline and in a strain expressing the three *P*. *somniferum* MTs producing (*S*)-reticuline. These experiments were used to demonstrate that the absence of salutartidine synthesis was due to the absence of (*R*)-reticuline production as opposed to poor PsSAS expression or activity. Results from the feeding experiments showed that cells expressing both PsSAS and PsCPR could catalyze the transformation of (*R*)-reticuline to salutaridine (Fig 4A and S1A Fig) and that (*S*)-reticuline was not consumed nor salutaridine produced in an (*S*)-reticuline-producing strain co-expressing the salutaridine synthase. It was therefore concluded that PsSAS is functional in yeast and that the enzyme is enantioselective for (*R*)-reticuline, as previously reported [19].

### Thebaine synthesis from salutaridine and (*R*)-reticuline

In opium poppy, salutaridine is converted to salutaridinol by the enzyme salutaridine reductase (PsSAR). PsSAR catalyzes the forward reaction converting salutaridine to salutaridinol at pH 6.0–6.5 and the reverse reaction at pH 9–9.5 [23]. When yeast cells expressing PsSAR were incubated with salutaridine at pH 8, the substrate was converted to salutaridinol demonstrating that the PsSAR is functional in yeast (S1B Fig).

Salutaridinol 7-*O*-acetyltransferase (PsSAT) acetylates salutaridinol to salutaridinol-7-*O*-acetate, which spontaneously rearranges to thebaine at pH 8–9 and to the side product dibenz[d,f]azonine at pH 6–7 [21,22]. Therefore, to determine if the pH of the feeding assay influenced thebaine yields when (*R*)-reticuline or salutaridine was used as substrate, the activity of our engineered thebaine-producing strains was tested at pH 7.5, 8, 8.5 and 9. To test the functional expression in yeast of the (*R*)-reticuline to thebaine pathway, cultures of strain GCY368 co-expressing PsSAS, PsCPR, PsSAR and PsSAT were incubated with (*R*)-reticuline or salutaridine as substrates. Thebaine was detected at all 4 pHs with a 3- and 4-fold higher yield at pH 8.5 and 9, respectively, than at pH 7.5 (Fig 4A and 4B). We observed the accumulation of the pathway intermediate salutaridine but not salutaridinol, salutaridinol-7-*O*-acetate or dibenz[d,f]azonine. These results confirmed functional expression of PsSAT and the spontaneous cyclization of salutaridino-7-*O*-acetate to thebaine, with an increasing efficiency using alkaline assay conditions. Accumulation of salutaridine when strain GCY368 is supplemented with either (*R*)-reticuline or salutaridine indicates that PsSAR is likely limiting flux through the recombinant thebaine-producing pathway (Fig 4).

### Synthesis of codeine and morphine in *S*. *cerevisiae*

**Fig 5:**
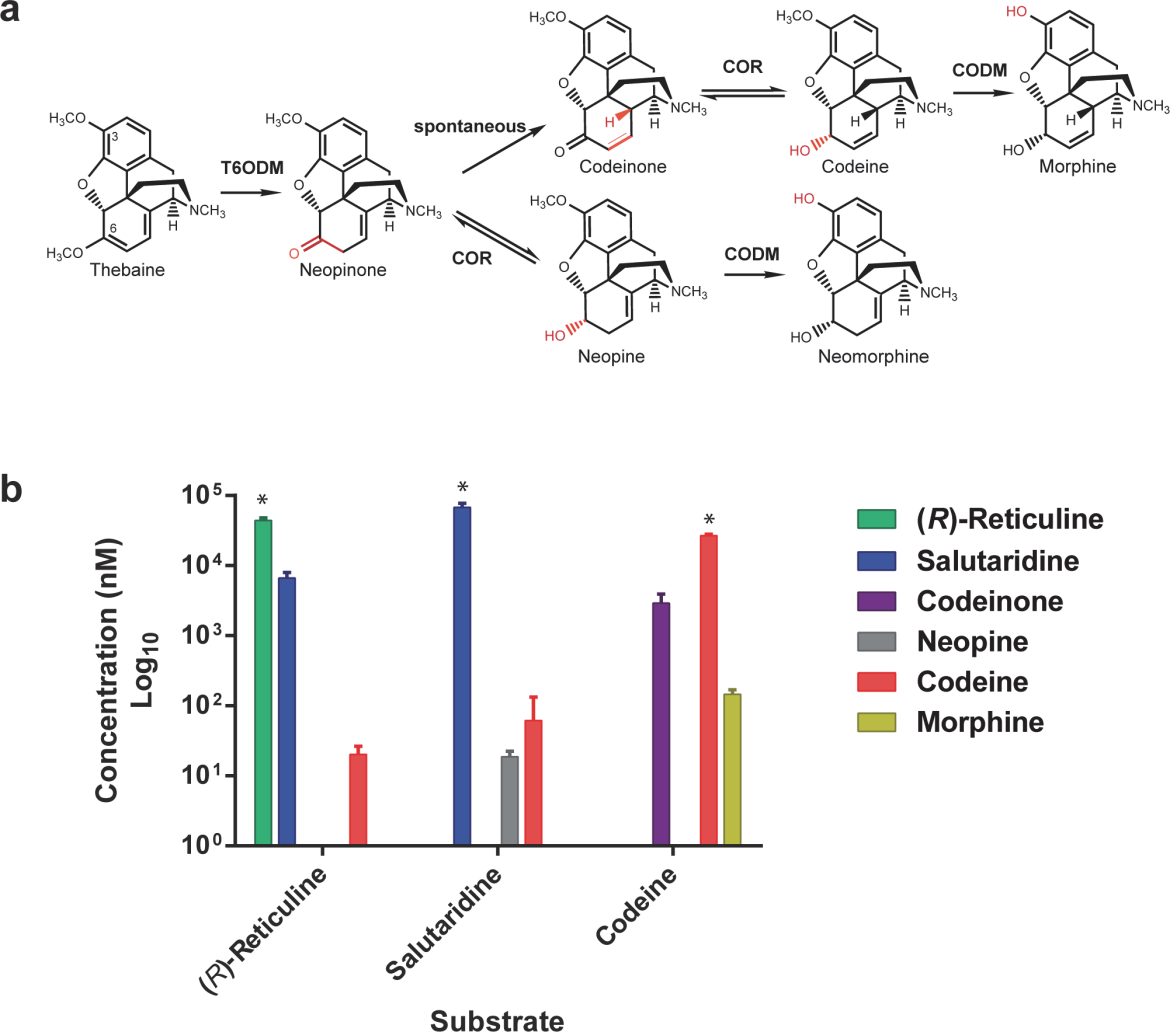
Synthesis of codeine and morphine in *S*. *cerevisiae*. **(a)** Schematic representation of the production of morphine from thebaine proceeding through the intermediate codeine. Thebaine is demethylated to neopinone by T6ODM. Neopinone spontaneously rearranges to codeinone or is reduced to the side product neopine by COR. Codeine and neopine are demethylated to morphine and the undesired side-product neomorphine by CODM. **(b)** Synthesis of codeine and morphine from whole cell feeding assays performed at pH 9 and supplemented with 100 μM (*R*)-reticuline, 100 μM salutaridine or 100 μM codeine. Strain GCY1358 was used in all cell feeding assays. Error bars represent standard deviations of n = 3. * Indicates the substrate used in the cell feeding assays.

Thebaine is the precursor to codeine and morphine synthesis (Fig 1) and to semi-synthetic opioids. Two possible pathways have been described for the production of morphine from thebaine, only one of which proceeds through the intermediate codeine (Fig 1). Thebaine 6-*O*-demethylase (PsT6ODM) and codeine demethylase (PsCODM) are the enzymes responsible of the demethylation steps at position 6 and 3, respectively. PsCODM can accept both thebaine and codeine, while PsT6ODM can demethylate both thebaine and oripavine [24]. The pathway proceeding through codeinone and codeine, which is the favorite route in yeast expressing the PsCODM, PsT6ODM and PsCOR, also leads to the side products neopine and neomorphine [5]. In this pathway, the codeinone reductase (PsCOR) reduces neopinone to neopine prior to the spontaneous rearrangement of neopinone to codeinone and neopine is demethylated to neomorphine by CODM (Fig 5A).

To test for a functional (*R*)-reticuline to morphine pathway in yeast and to identify potential bottlenecks, cells of strain GCY1358 co-expressing the 7 enzymes of the pathway (Fig 1) were supplemented with either (*R*)-reticuline, salutaridine or codeine and morphinan products were measured. Relative proportion of morphinan alkaloids was calculated as the total moles of alkaloid intermediate recovered from both cell extract and supernatant to moles of supplemented substrate. The accumulation of 15% salutaridine and 0.04% codeine was detected when (*R*)-reticuline was used as substrate (Fig 5B). When salutaridine was used as substrate, 0.1% codeine and 0.03% neopine were detected in the cell feeding assays (Fig 5A and 5B). No other opiate intermediates or side products were detected in the experimental condition tested. Since neither oripavine nor morphine was detected and both are products of the PsCODM, the functional expression of the CODM in strain GCY1358 using codeine as substrate was tested. In these assays, a conversion yield of codeine to morphine of 0.5%, was observed (Fig 5B) indicating that CODM is indeed functional in the engineered strain but its expression and/or activity are likely limiting flux to morphine synthesis. While intracellular transport of the supplemented codeine may also be a limiting factor, the absence of morphine synthesis from (*R*)-reticuline or salutaridine in strain GCY1358 is likely due to low efficiency of the overall pathway including CODM. Different factors could account for the observed low efficiency. A recent study on the synthesis of morphinan alkaloids from thebaine in *S*. *cerevisiae* showed that tuning gene copy number of PsT6ODM, PsCOR1.3 and PsCODM resulted in higher production of morphinan alkaloids, the preferred ratio of T6ODM:COR:CODM gene copies being 2:1:3 [5]. When codeine was supplemented to strain GCY1358, accumulation of 7% codeinone was also observed, indicating the non-productive oxidation of codeine by PsCOR (Fig 5A and 5B).

## Discussion

Synthesis of high-value plant specialized metabolites in microbes is emerging as an alternative to their extraction and purification from plants [25]. Microbial factories combine the power of enzymes in efficiently performing regio- and enantioselective reactions with the advantage of fast growing self-replicating systems that can be strictly controlled in a defined fermentation environment. Significant progress has been achieved in the synthesis of opiates using *S*. *cerevisiae* as a host [4,5]. In this work we demonstrate that synthesis of (*R*)-reticuline is an impediment to the reconstitution of a complete morphinan biosynthetic pathway in yeast. In addition, we show the functional expression of all known opium poppy genes leading to codeine and morphine synthesis in yeast and demonstrate for the first time that thebaine can be synthesized in *S*. *cerevisiae*. Thebaine and morphine are the two main opiates extracted from opium poppy latex, meaning that they are the starting precursors for the synthesis of other opioids [3]. Thebaine can be converted into a variety of bioactive pharmaceuticals including the opioid-antagonist naloxone and naltrexone and the analgesics oxycodone, oxymorphone, buprenorphine and etorphine.

Commercially available (*R*)-reticuline and salutaridine were used to test for the activity of the morphinan enzymes involved in the synthesis of salutaridine, salutaridinol and salutaridinol-7-*O*-acetate in *S*. *cerevisiae*. While the major route for the synthesis of (*R*)-reticuline in *P*. *somniferum* is considered to be epimerization from (*S*)-reticuline, the enzymes presumed to be involved in this reaction, DRS and DRR, have not been cloned [11,12]. It should however be noted that (*R*)-reticuline is not the only (*R*)-BIA intermediate found in Ranunculales [26]. This suggests the possibility of an alternative pathway for the synthesis of (*R*)-intermediates, possibly the existence of enzymes selective for the (*R*)-enantiomers from the very beginning of the reticuline synthesis pathway. For example, both (*S*)- and (*R*)-*N*-methylcoclaurine were isolated in *Berberis stolonifera*. These two enantiomers of *N*-methylcoclaurine are required by the cytochrome P450 berbamunine synthase for the synthesis of berbamunine in *Berberis stolonifera* [26]. While *P*. *somniferum* does not make (*R*,*S*)-norlaudanosoline, our results indicate that only (*S*)-reticuline is produced from racemic norlaudanosoline using opium poppy’s native methyltransferases. Some evidence for the enantioselectivity of MTs involved in BIA synthesis can be found in the literature. For example, a study reporting on the activity of *Coptis japonica* MTs for the production of reticuline from racemic norlaudanosoline in engineered *E*. *coli* reported a prevalent synthesis of (*S*)-reticuline over (*R*)-reticuline [18]. This data clearly indicated that some of the *C*. *japonica* MTs have a preference for the (*S*)-enantiomer with limited activity on the (*R*)-enantiomer. Combined, these results emphasize the importance of identifying a biosynthetic path to (*R*)-reticuline before the complete synthesis of opiates can be achieved in yeast.

Synthesis of thebaine from (*R*)-reticuline requires functional co-expression of four enzymes (Fig 1). While the existence of a thebaine synthase enzyme involved in the conversion of salutaridinol-7-*O*-acetate to thebaine can’t be excluded, an enzyme with this activity has not been isolated so far [21,27]. However, the intermediate salutaridinol-7-*O*-acetate can spontaneously rearrange to thebaine in solution at pH 8–9 and our results demonstrate that it does rearrange to thebaine in yeast cell feeding assays with a pH ranging from 7.5 to 9, which is likely equivalent to a maximum intracellular pH of ~7.5–8 [28]. Furthermore, although cellular metabolism is likely affected at an external pH above 8, enzymes involved in the synthesis of morphinan alkaloids appear to remain active, as demonstrated by thebaine production at pH>8 and viable plate counts showing no significant cell death after 16 hrs of incubation at pH 7.5, 8, 8.5 or 9 (S3 Fig).

Our results indicate that inefficient synthesis of salutaridinol from salutaridine is limiting flux to thebaine. This is illustrated by the fact that no difference in thebaine synthesis was observed between cell assays that were supplemented with 100 μM salutaridine (Fig 4B) and those that produced 10 μM salutaridine by using (*R*)-reticuline as a feeding substrate (Fig 4A). Intracellular transfer of substrate, poor PsSAR expression and/or catalytic properties could all contribute to the low efficiency of this conversion and should be investigated for pathway optimization. Salutaridine reductase from *Papaver bracteatum* (PbSAR), which differs only in 13 amino acids from PsSAR, is known to be substrate inhibited at low concentration of salutaridine (*K*_*i*_ = 150 μM) [29]. A previous mutagenesis study of PbSAR, based on homology modeling, resulted in identification of 2 mutants, F104A and I275A, with reduced substrate inhibition and increased *K*_*m*_, but slightly higher *k*_cat_. The double mutant F104A/I275A showed no substrate inhibition, with a higher *K*_*m*_ and *k*_cat_. Therefore, an increased flux through the (*R*)-reticuline to thebaine pathway could ostensibly be achieved by incorporating these mutations in PsSAR.

When the 7-gene morphine pathway was reconstituted in yeast we detected the formation of the expected metabolites with the exception of neopine (Fig 5A and 5B). The substrate promiscuity of the PsT6ODM, PsCODM and PsCOR is responsible for the accumulation of undesirable side-products such as those observed in the thebaine to morphine pathway [5]. Enzyme promiscuity in nature contributes to the great chemo-diversity of plant specialized metabolites [30] and could be exploited to produce a variety of new molecules. However, this same property leads to the formation of undesirable side-products when pathway reconstitution is used to produce targeted metabolites [4]. PsCODM, PsT6ODM and PsCOR are all great examples of enzyme promiscuity [24,31,32] but they may become a significant problem for the synthesis of targeted BIAs in yeast as they are known to have broad substrate specificity [31]. While plants use cellular compartmentalization of competing activities and transport to channel specific syntheses towards specialized cell types [33] and tissue, this represents a challenging engineering problem in microorganisms. Solutions could be to generate synthetic microbial compartments [34], multi-enzyme scaffolds to channel intermediates to the pathway of interest [35] or alteration of an enzyme’s specificity by protein engineering or by identifying orthologs/paralogs from plant transcriptome databases [36].

While major challenges remain to be addressed for the viable synthesis of opiates in yeast, like the production of BIAs from simple carbon sources and the identification of the enzymes involved in the synthesis of (*R*)-intermediates, this study provides an important advancement on the synthesis of morphinan alkaloids in yeast. We demonstrate the functional expression in *S*. *cerevisiae* of all seven enzymes involved in the synthesis of morphine from (*R*)-reticuline at alkaline pH and the importance of developing pH-adaptable fermentation conditions for synthesis of opiates in yeast.

## Supporting Information

S1 Fig Functional activity of PsSAS, PsCPR and PsSAR in *S. cerevisiae*. LC-FT-MS chromatographic profile of culture supernatants from cell feeding assays of **(a)** strain GCY1356 encoding for PsSAS and PsCPR and incubated with 100 μM (*R*)-reticuline and **(b)** strain GCY258 encoding for PsSAR and incubated with 100 μM salutaridine. Extracted ion chromatograms for *m/*z = 328 and *m/*z = 330 confirm the production of salutaridine and salutaridinol, respectively.
(TIF)

S2 Fig Extracted ion chromatograms and MS2 spectrum of intermediates accumulated by *S*. *cerevisiae* expressing the reticuline to morphine pathway. Cell feeding assays using **(a)** 100 μM (*R*)-reticuline; **(b)** 100 μM salutaridine or **(c)** 100 μM codeine. S corresponds to salutaridine; C corresponds to codeine; N corresponds to neopine; CC corresponds to codeinone; M corresponds to morphine.
(TIF)

S3 Fig Viable plate count for cell feeding assays. *S*. *cerevisiae* plate count assays showing cell viability before (time 0) and after (time 16 hrs) incubation in a Tris-HCl buffer at a pH ranging from 7.5 to 9. Bars represent a range of n = 2.
(TIF)

S1 Table List of *Saccharomyces cerevisiae* strains and plasmids used in this study. (DOCX)

S2 Table Common regions used for cloning. (DOCX)

S3 Table Oligonucleotides used for amplification of expression construct parts. (DOCX)
