# Supplementary figures and images for: Synthesis of Morphinan Alkaloids in Saccharomyces cerevisiae
Source: PLoS One. 2015 Apr 23;10(4):e0124459. doi: 10.1371/journal.pone.0124459 (PMC4408053; doi:10.1371/journal.pone.0124459)

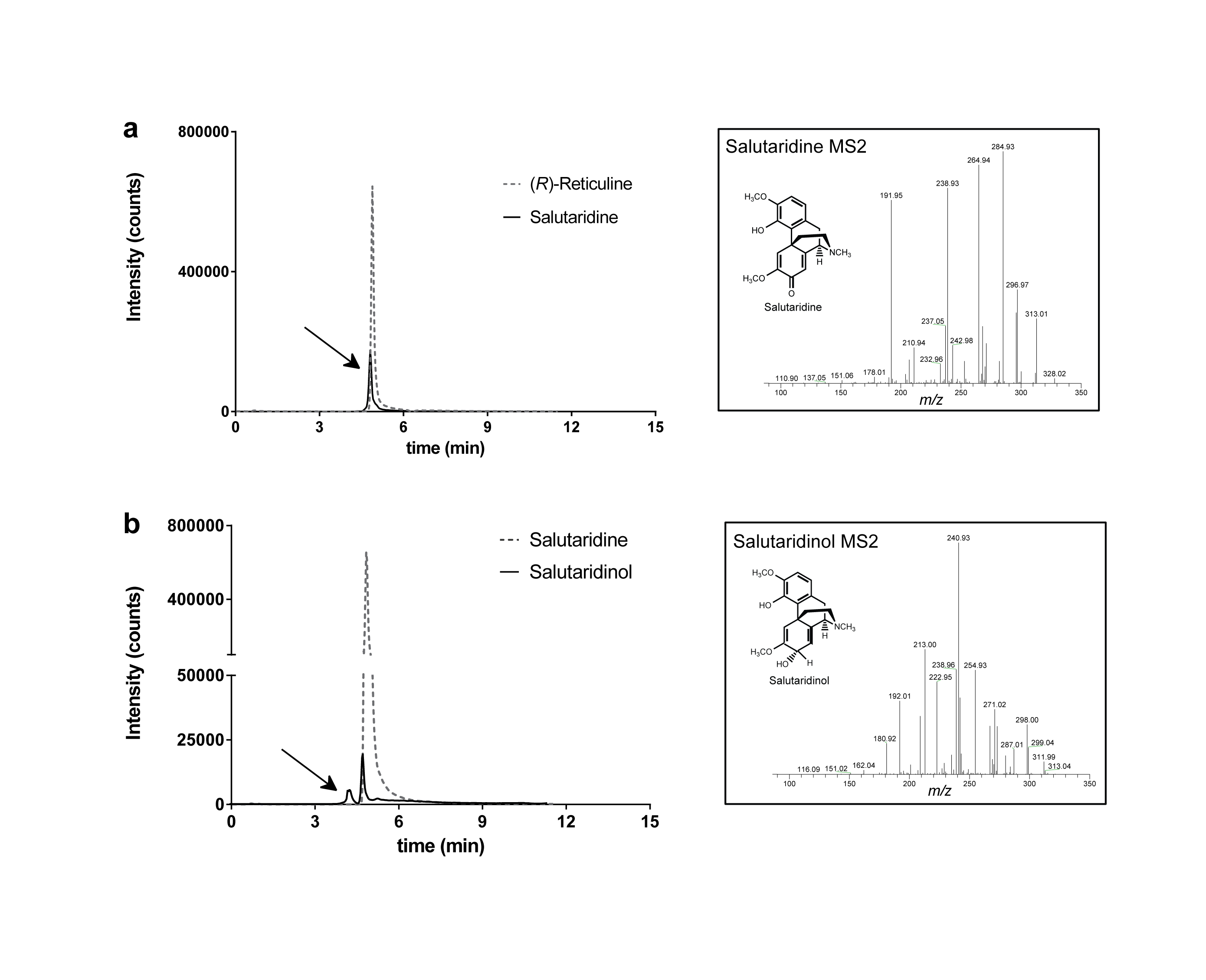

Supplement: S1 Fig — LC-FT-MS chromatographic profile of culture supernatants from cell feeding assays of (a) strain GCY1356 encoding for PsSAS and PsCPR and incubated with 100 μM (R)-reticuline and (b) strain GCY258 encoding for PsSAR and incubated with 100 μM salutaridine. Extracted ion chromatograms for m/z = 328 and m/z = 330 confirm the production of salutaridine and salutaridinol, respectively. (TIF) [file pone.0124459.s001.tif]

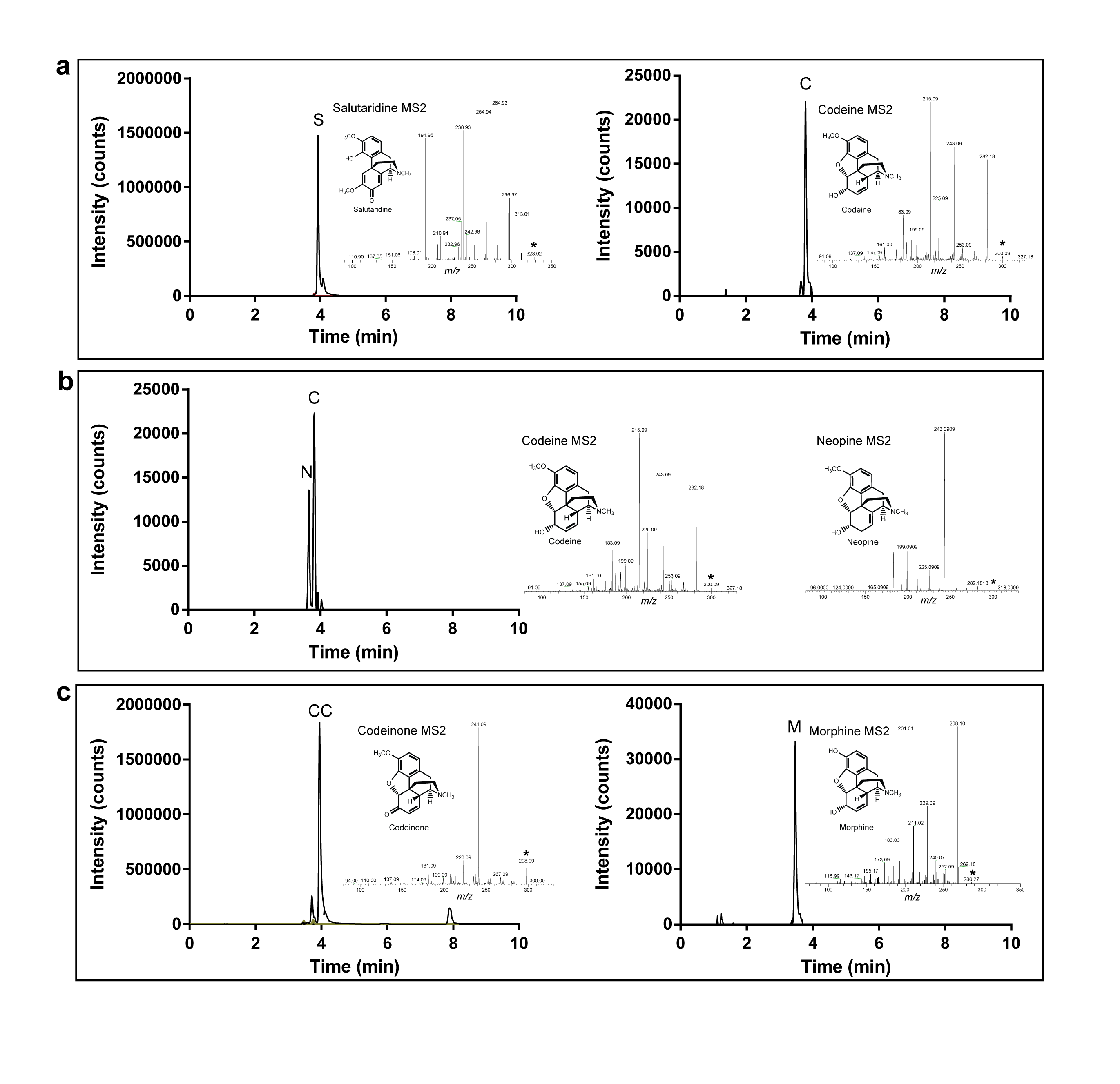

Supplement: S2 Fig — Cell feeding assays using (a) 100 μM (R)-reticuline; (b) 100 μM salutaridine or (c) 100 μM codeine. S corresponds to salutaridine; C corresponds to codeine; N corresponds to neopine; CC corresponds to codeinone; M corresponds to morphine. (TIF) [file pone.0124459.s002.tif]

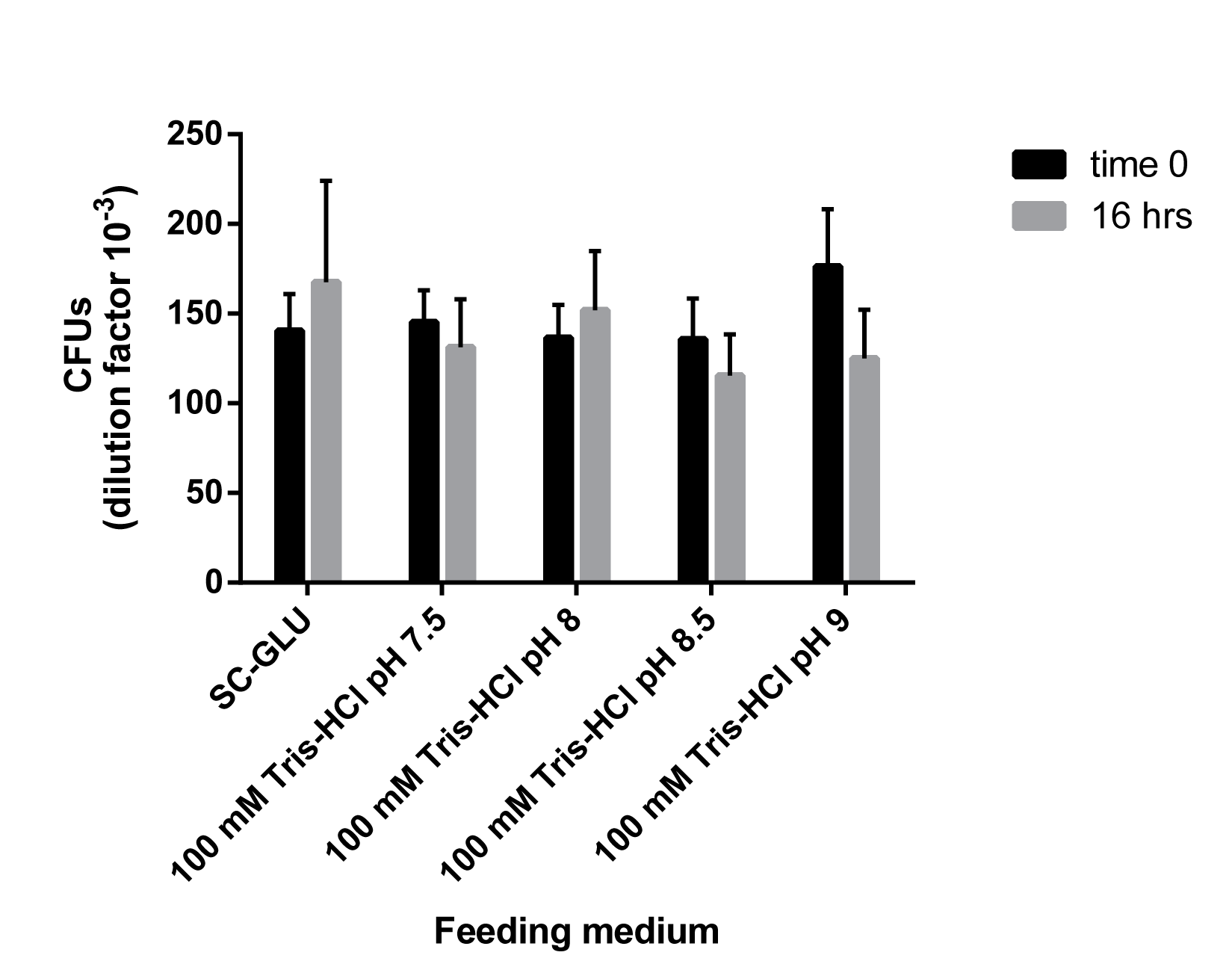

Supplement: S3 Fig — S. cerevisiae plate count assays showing cell viability before (time 0) and after (time 16 hrs) incubation in a Tris-HCl buffer at a pH ranging from 7.5 to 9. Bars represent a range of n = 2. (TIF) [file pone.0124459.s003.tif]
