# Supplementary material for: Synthesis of Morphinan Alkaloids in Saccharomyces cerevisiae
Source: PLoS One. 2015 Apr 23;10(4):e0124459. doi: 10.1371/journal.pone.0124459 (PMC4408053; doi:10.1371/journal.pone.0124459)
Supplement: S1 Table — (DOCX) [file pone.0124459.s004.docx]

**Table S1. List of *Saccharomyces cerevisiae* strains and plasmids used in this study.**

| Strain | Genotype | Plasmid | Source |
| --- | --- | --- | --- |
| CEN.PK113-13D | *MAT*α *ura3-52 MAL2-8C SUC2* | none | [31] |
| CEN.PK110-16D | *MAT*α *trp1-289 MAL2-8C SUC2* | none | [31] |
| CEN.PK2-1D | *MATα ura3-52 trp1-289 leu2-3,112 his3-Δ1 MAL2-8C SUC2* |  | [31] |
| CEN.PK113-16B | *MAT*α *leu2-3 MAL2-8C SUC2* | none | [31] |
| CEN.PK110-10C | *MAT*α *his3* Δ*1 MAL2-8C SUC2* | none | [31] |
| CEN.PK113-14C | *MAT*α *leu2-3,112 his3* Δ*1 MAL2-8C SUC2* | none | [31] |
| CEN.PK110-7C | *MAT*α *ura3-52 trp1-289 MAL2-8C SUC2* | none | [31] |
| CEN.PK113-17A | *MAT*α *ura3-52 leu2-3,112 MAL2-8C SUC2* | none | [31] |
| GCY256 | CEN.PK113-13D | pGC263 (SAS-HA tag) | This study |
| GCY257 | CEN.PK113-13D | pGC264 (CPR-HA tag) | This study |
| GCY258 | CEN.PK113-13D | pGC265 (SAR-HA tag) | This study |
| GCY368 | CEN.PK113-16D | pGC359 (SAS,CPR,SAR,SAT) | This study |
| GCY1082 | CEN.PK2-1D *YORWΔ17(ChrXV)::***C1**-*P_TDH3_*-*PsCFS*-*T_CYC1_*-**C6-H1-C1**-*P_PDC1_*-*PsBBEΔN*-*T_ADH1_*-**C6-H2-C1**-*P_PMA1_-PsSPSΔN^b^-T_PGI1_-***C6**; *YPRCΔ15(ChrXVI)::***C1**-*P_PDC1_*-*P6H*-*T_CYC1_*-**C6-H1-C1**-*P_TDH3_*-*MSH-T_ADH1_*-**C6-C1**-*P_FBA1_-TNMT-T_PGI1_-***C6** | none | [8] |
| GCY1086 | CEN.PK113-16B | pGC1062 (6OMT,4’OMT,CNMT) | [8] |
| GCY1125 | GCY1082 (dihydrosanguinarine producing strain) | pGC1062 (6OMT,4’OMT,CNMT); pGC557 (CPR); pGC655 (BBE) | [8] |
| GCY1356 | CEN.PK110-10C | pGC719 (SAS, CPR) | This study |
| GCY1357 | CEN.PK113-14C | pGC1062 (6OMT, CNMT, 4’OMT); pGC719 (SAS, CPR) | This study |
| GCY1358 | CEN.PK110-7C | pGC359 (SAS, CPR, SAR, SAT); pGC11 (CODM, T6OMD, COR) | This study |
| GCY1359 | CEN.PK113-17A | pGC1062 (6OMT, CNMT, 4’OMT); pGC655 (BBE) | This study |
| Plasmid name | **Genotype ^a, b^** |  | **Source** |
| pYES2 | 2μ^ori^, *pUC*^ori^, *URA3, Amp*^R^, *P_GAL1_-T_CYC1_* | | Invitrogen |
| pGC263 | *pYES2::P_GAL1_-PsSAS-HA tag – T_CYC1_* | | This study |
| pGC264 | *pYES2::P_GAL1_-PsCPR-HA tag – T_CYC1_* | | This study |
| pGC265 | *pYES2::P_GAL1_-PsSAR-HA tag – T_CYC1_* | | This study |
| pGREG503 | CEN6/ARS4^ori^*, pMB1*^ori^, *HIS3, Amp*^R^*, loxP-Kan*^R^, *P_GAL1_-HISstuffer-T_CYC1_* | | [28] |
| pGREG504 | CEN6/ARS4^ori^, *pMB1^ori^*, *TRP1, Amp*^R^*, loxP-Kan*^R^, *P_GAL1_-HISstuffer-T_CYC1_* | | [28] |
| pGREG505 | CEN6/ARS4^ori^, *pMB1^ori^*, *LEU2, Amp*^R^*, loxP-Kan*^R^, *P_GAL1_-HISstuffer-T_CYC1_* | | [28] |
| pGREG506 | CEN6/ARS4^ori^*, pMB1^ori^*, *URA3, Amp*^R^*, loxP-Kan*^R^, *P_GAL1_-HISstuffer-T_CYC1_* | | [28] |
| pGC964 | *pGREG503 ΔKpnI^(3555-2560)^A(3558)G*, Δ*KpnI^(4509-4514)^ A(4512)G* | | [8] |
| pGC965 | *pGREG504* Δ*KpnI^(3555-2560)^A(3558)G* | | [8] |
| pGC966 | *pGREG505* Δ*KpnI^(3555-2560)^A(3558)G*, Δ*KpnI^(5176-5181)^ A(5179)G* | | [8] |
| pGC967 | *pGREG506* Δ*KpnI^(3593-3598)^A(3596)G* | | [8] |
| pGC359 | *pGC965::***C1**-*P_TEF2_*-*PsSAS*-**C4-H15-C5**-*T_CYC1_*-**C6-H1-C1**-*P_PDC1_*-*PsCPR***-C4-H14-C5-***T_TDH2_*-**C6-H2-C1**-*P_FBA1_-PsSAT***-C4-H12-C5-***T_ADH1_***-C6-H3-C1-** *P_TDH3_-PsSAR***-C4-H16-C5-***T_PGI1_***-C6** | | This study |
| pGC1062 | *pGC966::***C1**-*P_TDH3_*-*Ps6OMT*-*T_CYC1_*-**C6-H1-C1**- *P_FBA1_*-*Ps4’OMT*-*T_ADH1_*-**C6-H2-C1**- *P_PDC1_-PsCNMT-T_PGI1_-***C6** | | [8] |
| pGC557 | *pGC964::***C1**-*P_TDH3_*-*PsCPR*-*T_CYC1_*-**C6** | | [8] |
| pGC655 | *pYES2::P_PMA1_-PsBBEΔN-T_PGI1_* | | [8] |
| pGC719 | pGC964*::***C1**-*P_FBA1_*-*PsCPR*-*T_CYC1_*-**C6-H1-C1-** *P_PMA1_*-*PsSAS*-*T_ADH1_*-**C6** | | This study |
| pGC11 | pGC967*::P_PGK1_*-*PsT6ODM*-*T_CYC1_*-*P_TPI1_*-*PsCODM***-***T_ADH1_*-*P_TEF1_-PsCOR***-***T_PGI1_* | | This study |

^a^ All the genes used in this study are synthetic genes and sequences were codon-optimized for expression in *Saccharomyces cerevisiae*.

^b^ Linkers used for cloning purposes are in bold.
