# Supplementary material for: Synthesis of Morphinan Alkaloids in Saccharomyces cerevisiae
Source: PLoS One. 2015 Apr 23;10(4):e0124459. doi: 10.1371/journal.pone.0124459 (PMC4408053; doi:10.1371/journal.pone.0124459)
Supplement: S2 Table — (DOCX) [file pone.0124459.s005.docx]

**Table S2. Common regions used for cloning purposes.**

| **Primer name** | **Sequence 5’→3’** |
| --- | --- |
| C1 | GAGACTGCAGCATTACTTTGAGAAG |
| C2 | GTCCAGAGTCAGTGTGTATCTACT |
| C3 | GTTGTCAGCAACGACGATATCTG |
| C4 | GGCTACTTCGGTGTACCAAACTAA |
| C5 | TCACTTACACGAGGAGATGCATTG |
| C6 | GGCAATCACATCACCATGAGTTGT |
| H1 | CTCATGGCGGGGGTCGGAATGATTAAAGAAAGGGGCTGTGGGCGAGATTG |
| H2 | CCAGTTAATAAACCGTGGCAAACATGATGGTGGCCTAATGGAGGTCACCA |
| H3 | ATTTTACAACCAGAACACAAAAGTGCGAAGTTTGAGCAACGGCGACGGAT |
| H12 | GAGCGTAGGTTCCAAGATCCCCAGTTCAAAAGGATCCGTTCTAGTGCCAG |
| H14 | TTATGAACCGTGTGTGACCCTTTCCGAGCGAGTGTTGGGGTTCCACGCTC |
| H15 | ACTTCTGCGCTGTACCCGTGGTAATACTTGTCACCTTAGTTTGCGATAAC |
| H16 | ACCTAATGGTTCTTCGTTGCTATAGCAGGTGGCAGGGACCCAACATCATA |
