# Supplementary material for: Synthesis of Morphinan Alkaloids in Saccharomyces cerevisiae
Source: PLoS One. 2015 Apr 23;10(4):e0124459. doi: 10.1371/journal.pone.0124459 (PMC4408053; doi:10.1371/journal.pone.0124459)
Supplement: S3 Table — (DOCX) [file pone.0124459.s006.docx]

**Table S3. Oligonucleotides used for amplification of expression construct parts.**

| Name | Sequence 5’→3’ | |
| --- | --- | --- |
| pYES2_backbone | | |
| pYES2 for | | TACCCATACGATGTTCCAGATTACGCTTAAATCATGTAATTAGTTATGTCACGCTTAC |
| pYES2 rev | | CTCCTTGACGTTAAAGTATAGAGG |
| pGC263 (SAS-HA-tag) | | |
| SAS HA tag for | | ATATACCTCTATACTTTAACGTCAAGGAGAAAACAATGGCACCAATCAACATTGAAG |
| SAS HA tag rev | | TTAAGCGTAATCTGGAACATCGTATGGGTATTGACGAAATGGTTTAGTACGTGGAG |
| pGC264 (CPR-HA tag) | | |
| CPR HA Tag F | | ATATACCTCTATACTTTAACGTCAAGGAGAAAACAATGGGGTCAAACAACCTGG |
| CPR HA Tag R | | TTAAGCGTAATCTGGAACATCGTATGGGTACCATACATCTCTCAAGTATCTCTC |
| pGC265 (SAR-HA tag) | | |
| SAR-HA tag for | | ATATACCTCTATACTTTAACGTCAAGGAGAAAACAATGCCAGAAACTTGTCCAAATACGG |
| SAR-HA tag rev | | TTAAGCGTAATCTGGAACATCGTATGGGTAGAAGGCGGACAACTCAGAACAATC |
| pGC359 (thebaine block) | | |
| pG:C1 for | | TAACCCTCACTAAAGGGAACAAAAGCTGGAGCTCGTTTAAACGGCGCGCCGAGACTGCAGCATTACTTTGAGAAG |
| TEF2p rev | | CTTCAATGTTGATTGGTGCCATTGTTTTTTAATTATAGTTCGTTGACCGTATATTC |
| SAS for | | TTAGAATATACGGTCAACGAACTATAATTAAAAAACAATGGCACCAATCAACATTGAAG |
| C4:H15 rev | | GTTATCGCAAACTAAGGTGACAAGTATTACCACGGGTACAGCGCAGAAGTTTAGTTTGGTACACCGAAGTAGCC |
| C5:H15for | | ACTTCTGCGCTGTACCCGTGGTAATACTTGTCACCTTAGTTTGCGATAACTCACTTACACGAGGAGATGCATTG |
| C6:H1 rev | | CAATCTCGCCCACAGCCCCTTTCTTTAATCATTCCGACCCCCGCCATGAGACAACTCATGGTGATGTGATTGCC |
| C1:H1 frw | | CTCATGGCGGGGGTCGGAATGATTAAAGAAAGGGGCTGTGGGCGAGATTGGAGACTGCAGCATTACTTTGAGAAG |
| PDC1p rev | | CCAGGTTGTTTGACCCCATTGTTTTTGATTTGACTGTGTTATTTTGCGTGAG |
| CPR for | | CCAGGTTGTTTGACCCCATTGTTTTTGATTTGACTGTGTTATTTTGCGTGAG |
| C4:H14 rev | | GAGCGTGGAACCCCAACACTCGCTCGGAAAGGGTCACACACGGTTCATAATTAGTTTGGTACACCGAAGTAGCC |
| C5:H14 for | | TTATGAACCGTGTGTGACCCTTTCCGAGCGAGTGTTGGGGTTCCACGCTCTCACTTACACGAGGAGATGCATTG |
| C6:H2 rev | | TGGTGACCTCCATTAGGCCACCATCATGTTTGCCACGGTTTATTAACTGGACAACTCATGGTGATGTGATTGCC |
| C1:H2 for | | CCAGTTAATAAACCGTGGCAAACATGATGGTGGCCTAATGGAGGTCACCAGAGACTGCAGCATTACTTTGAGAAG |
| FBA1p rev | | GCAGAATACATTGTGGCCATTGTTTTTATGTATTACTTGGTTATGGTTATATATGAC |
| SAT for | | GTCATATATAACCATAACCAAGTAATACATAAAAACAATGGCCACAATGTATTCTGC |
| C4:H12 rev | | CTGGCACTAGAACGGATCCTTTTGAACTGGGGATCTTGGAACCTACGCTCTTAGTTTGGTACACCGAAGTAGCC |
| C5:H12 for | | GAGCGTAGGTTCCAAGATCCCCAGTTCAAAAGGATCCGTTCTAGTGCCAGTCACTTACACGAGGAGATGCATTG |
| C6:H3 rev | | ATCCGTCGCCGTTGCTCAAACTTCGCACTTTTGTGTTCTGGTTGTAAAATACAACTCATGGTGATGTGATTGCC |
| C1:H3 for | | ATTTTACAACCAGAACACAAAAGTGCGAAGTTTGAGCAACGGCGACGGATGAGACTGCAGCATTACTTTGAGAAG |
| TDH3p rev | | GTATTTGGACAAGTTTCTGGCATTGTTTTTCGAAACTAAGTTCTTGGTGTTTTAAAAC |
| SAR_for | | GTTTTAAAACACCAAGAACTTAGTTTCGAAAAACAATGCCAGAAACTTGTCCAAATAC |
| C4:H16 rev | | TATGATGTTGGGTCCCTGCCACCTGCTATAGCAACGAAGAACCATTAGGTTTAGTTTGGTACACCGAAGTAGCC |
| C5:H16 for | | ACCTAATGGTTCTTCGTTGCTATAGCAGGTGGCAGGGACCCAACATCATATCACTTACACGAGGAGATGCATTG |
| pG:C6 rev | | ATAACTTCGTATAATGTATGCTATACGAAGTTATTAGGTACCGCGGCCGCACAACTCATGGTGATGTGATTGCC |
| pGC719 (SAS, CPR) | |  |
| pG:C1 for | | TAACCCTCACTAAAGGGAACAAAAGCTGGAGCTCGTTTAAACGGCGCGCCGAGACTGCAGCATTACTTTGAGAAG |
| FBA1p rev | | GCAGAATACATTGTGGCCATTGTTTTTATGTATTACTTGGTTATGGTTATATATGAC |
| FBA1p for | | GCCAGGTTGTTTGACCCCATTGTTTTTATGTATTACTTGGTTATGGTTATATATGAC |
| CPR for | | GTCATATATAACCATAACCAAGTAATACATAAAAACAATGGGGTCAAACAACCTGGC |
| CPR rev | | GTAAGCGTGACATAACTAATTACATGATTACCATACATCTCTCAAGTATCTCTC |
| CYC1t for | | GAGAGATACTTGAGAGATGTATGGTAATCATGTAATTAGTTATGTCACGCTTAC |
| C6:H1 rev | | CAATCTCGCCCACAGCCCCTTTCTTTAATCATTCCGACCCCCGCCATGAGACAACTCATGGTGATGTGATTGCC |
| C1:H1 for | | CTCATGGCGGGGGTCGGAATGATTAAAGAAAGGGGCTGTGGGCGAGATTGGAGACTGCAGCATTACTTTGAGAAG |
| PMA1p rev | | GTTGATTGGTGCCATTGTTTTTTTGATAATTAAATCTTTCTTATCTTCTTATTCTTTTC |
| SAS for | | GATAAGAAAGATTTAATTATCAAAAAAACAATGGCACCAATCAACATTGAAGGTAAC |
| SAS rev | | GAGACTTGACCAAACCTCTGGCGAAGAAGTCCACTATTGACGAAATGGTTTAGTACGTG |
| ADH1t for | | GATAACTCCACGTACTAAACCATTTCGTCAATAGTGGACTTCTTCGCCAGAGGTTTG |
| pYES2:C6 rev | | CAATACGCAAACCGCCTCTCCCCGCGCGTTGGCCGATTCATTAATGCAGGACAACTCATGGTGATGTGATTGCC |
| pGC11 (morphine block) | | |
| PG:PGK1p for | | TAACCCTCACTAAAGGGAACAAAAGCTGGAGCTCGTTTAAACGGCGCGCCACGCACAGATATTATAACATCTGCATAATA |
| PGK1p rev | | TCATCAGTTTAGCTTTCTCCATTGTTTTATATTTGTTGTAAAAAGTAGATAATTACTTCCTTG |
| T6ODM for | | CAAGGAAGTAATTATCTACTTTTTACAACAAATATAAAACAATGGAGAAAGCTAAACTGATGA |
| T6ODM rev | | GAATGTAAGCGTGACATAACTAATTACATGATCAAATACGCATAGAATCTAGAAATGATTTTC |
| CYC1t for | | GAAAATCATTTCTAGATTCTATGCGTATTTGATCATGTAATTAGTTATGTCACGCTTACATTC |
| CYC1 rev | | CTAAGTAAGTTAAATATCCGTAATCTTTAAACAGCTAGTGCAAATTAAAGCCTTCGAGCGTC |
| TPI1 for | | GTTTTGGGACGCTCGAAGGCTTTAATTTGCACTAGCTGTTTAAAGATTACGGATATTTAAC |
| TPI1 rev | | GCTTGATTAAGATAGGAGTTTCCATTGTTTTTTTTAGTTTATGTATGTGTTTTTTGTAGTTATAG |
| CODM for | | CTATAACTACAAAAAACACATACATAAACTAAAAAAAACAATGGAAACTCCTATCTTAATCAAGC |
| CODM rev | | CTTGACCAAACCTCTGGCGAAGAAGTCCATTACATTCTCATATAGTCTAGGAAAGATTTC |
| ADH1t for | | CTTGATGGGAAATCTTTCCTAGACTATATGAGAATGTAATGGACTTCTTCGCCAGAGGTTT |
| ADH1t rev | | TCTGGAAGAGTAAAAAAGGAGTAGAAACATTTTGAAGCTATGCATGCCGGTAGAGGTGTG |
| TEF1p for | | CTTATTGACCACACCTCTACCGGCATGCATAGCTTCAAAATGTTTCTACTCCTTTTTTAC |
| TEF1p rev | | ATTGGGACGCCATTAGATTCCATTGTTTTATTAAAACTTAGATTAGATTGCTATGCTTTCT |
| COR for | | GAAAGAAAGCATAGCAATCTAATCTAAGTTTTAATAAAACAATGGAATCTAATGGCGTCCC |
| COR rev | | TGTTCTTTAGGTATATATTTAAGAGCGATTTGTTTTAATCTTTTTCATCCCAGAACTCCTC |
| PGI1t for | | GAGGAGTTCTGGGATGAAAAAGATTAAAACAAATCGCTCTTAAATATATACCTAAAGAACA |
| pG:PGI1t rev | | ATAACTTCGTATAATGTATGCTATACGAAGTTATTAGGTACCGCGGCCGCGGTATACTGGAGGCTTCATGAG |

|  |  |
| --- | --- |
